# rTMS in mental health disorders

**DOI:** 10.3389/fnetp.2023.943223

**Published:** 2023-07-28

**Authors:** Kneginja Richter, Stefanie Kellner, Christiane Licht

**Affiliations:** ^1^ Paracelsus Medical Private University, Nuremberg, Germany; ^2^ Department for Social Sciences, Georg Simon Ohm University of Applied Sciences Nuremberg, Nuremberg, Germany; ^3^ Faculty of Medical Sciences, Goce Delcev University, Stip, North Macedonia

**Keywords:** neurostimulation, depression, catatonia, sleep, electroencephalogram, insomnia, repetitive transcranial magnetic stimulation

## Abstract

Transcranial magnetic stimulation (TMS) is an innovative and non-invasive technique used in the diagnosis and treatment of psychiatric and neurological disorders. Repetitive TMS (rTMS) can modulate neuronal activity, neuroplasticity and arousal of the waking and sleeping brain, and, more generally, overall mental health. Numerous studies have examined the predictors of the efficacy of rTMS on clinical outcome variables in various psychiatric disorders. These predictors often encompass the stimulated brain region’s location, electroencephalogram (EEG) activity patterns, potential morphological and neurophysiological anomalies, and individual patient’s response to treatment. Most commonly, rTMS is used in awake patients with depression, catatonia, and tinnitus. Interestingly, rTMS has also shown promise in inducing slow-wave oscillations in insomnia patients, opening avenues for future research into the potential beneficial effects of these oscillations on reports of non-restorative sleep. Furthermore, neurophysiological measures emerge as potential, disease-specific biomarkers, aiding in predicting treatment response and monitoring post-treatment changes. The study posits the convergence of neurophysiological biomarkers and individually tailored rTMS treatments as a gateway to a new era in psychiatric care. The potential of rTMS to induce slow-wave activity also surfaces as a significant contribution to personalized treatment approaches. Further investigations are called for to validate the imaging and electrophysiological biomarkers associated with rTMS. In conclusion, the potential for rTMS to significantly redefine treatment strategies through personalized approaches could enhance the outcomes in neuropsychiatric disorders.

## Introduction

Transcranial magnetic stimulation (TMS) is a non-invasive method that modulates neural processing in the brain by stimulating a magnetic coil that generates a magnetic field which then induces depolarizing neuronal cell membrane potentials in the cortical tissue beneath the coil and influences the associated activity of neural loops. The context-dependent overall number of pulses, the location of the coil in relation to the brain, the frequency and intensity of the magnetic stimulation, the time duration between each string, and the target regions on the cortex are linked to the effects of TMS (Pashut et al., 2011; Reithler et al., 2011; Peng et al., 2018). The mechanism of repetitive TMS (rTMS) is based on increasing neuronal activity, enhancing synaptic connectivity of neurons, strengthening local blood circulation, increasing oxygen consumption, and probably triggering neuroplasticity in the stimulated area (Pashut et al., 2011). Research over the last century using rTMS resulted in scientific studies indicating the effectiveness of rTMS for many psychiatric and neurological conditions, subsequently leading to the evidence-based studies that resulted in rTMS being included in the guidelines as an effective treatment for depression (Wassermann, 1998; George et al., 1999; Lefaucheur et al., 2014).

Moreover, there are a growing number of studies supporting the efficacy of rTMS for other conditions such as insomnia, catatonia, post-traumatic stress disorder, anxiety, Parkinson’s disease, tinnitus, MCI, and other diagnoses (Ladenbauer et al., 2017; Richter et al., 2017; Licht et al., 2021; Li et al., 2022; Li et al., 2022; Kan et al., 2023). Nevertheless, rTMS outcomes have also been reported as heterogeneous with studies suggesting subpopulations of fast and slow responders as well as non-responders (O’Reardon et al., 2007; Reithler et al., 2011; Yip et al., 2017; Lefaucheur et al., 2020).

Most of the studies were conducted during the waking state. At the same time, positive effects on the consolidation of neuronal activity were found in the polysomnographic recordings, suggesting a positive influence on sleep quality.

Despite the proven effects of rTMS at the neuronal level and having a positive impact on clinical outcome variables, the question about the cause of non-response in some patients remains unanswered. An answer can be sought in the analysis of the following conditions: 1) the stimulation protocols vary between research groups, 2) the frequency used has a different effect on different individuals, 3) the timing of the rTMS could show a different effect depending on day or night when the stimulation was applied, 4) the electroencephalogram (EEG) activity of the stimulated areas shows chronobiological oscillations that could play a role in potentiating or inhibiting the EEG activity of the stimulated brain region, 5) the pathology of the disease could be located elsewhere than in the stimulated region, and 6) the neuronal connectivity of the stimulated region could be primed by the previous treatment that suppresses the reactivity of the neurons underlying the coil, such as polypharmacotherapy, and for other unknown reasons.

Traditional TMS studies report on the impact of TMS on neural activity in specific brain regions and its impact on underlying connectivity and its alterations. Therefore, it is important to perform targeted measurements of functional connectivity and/or neural network and investigate how connectivity and network changes occur (Arns et al., 2012).

Changes in the amplitude of motor evoked potentials (MEPs) elicited by transcranial magnetic stimulation are a suitable model to study the neurophysiology of rTMS and its effects on the brain, i.e., different ways in which rTMS stimulation protocols can be used therapeutically. The interindividual variability of the mean MEP response to rTMS has been investigated in many studies, leading to questions about the utility of this model. rTMS is able to modulate individual moment-to-moment variability in neuronal activity, and this may have implications for the therapeutic application of rTMS (Goldsworthy et al., 2021).

The aim of this review is to use a non-standardized approach to measuring rTMS-induced neuroplasticity, including stimulation in the evening and the effects of rTMS on the sleep–wake rhythm of treated subjects.

## Methods

A literature search was conducted in March 2022 using the scientific databases PubMed and Google Scholar, covering publications from January 2003 to March 2022. Studies were identified by combinations of the following keywords in the title or abstract: rTMS, neuroplasticity, individual differences, variability, clinical predictors, sleep–wake rhythm, sleep, and EEG.

The literature review was limited to published research articles written in English. In addition, to be included in this review, the identified articles had to be original studies or reviews on the effects of rTMS on various disorders and on possible clinical predictors or correlates of treatment outcomes when using rTMS. First, studies whose titles clearly indicated that they were unrelated to the topic were excluded. The abstracts of the remaining studies were then analyzed for relevance to the topic, which led to a second wave of exclusions. After exclusion at the title and abstract level, the remaining articles were thoroughly analyzed in full text, as well as their reference list screened, to identify relevant publications that were not covered by the search strategy. Ultimately, 17 studies that met the previously defined inclusion criteria were included in the literature review.

## Results

### rTMS in depression

For the treatment of depressive disorders, the left or right dorsolateral prefrontal cortex (DLPFC) is often the focus of repeated stimulation employed daily for 3–6 weeks by a strong, time-varying magnetic field (pulse duration 100–400 μs and intensity 1.5–2.5 Tesla) (Wassermann, 1998). This approach, based on imaging studies, emerged as a novel antidepressant treatment, with multiple studies corroborating the acute antidepressant effects of high-frequency (10–20 Hz) rTMS of the left DLPFC. Among these studies, two large multicenter trials demonstrated that a monotherapy high-frequency rTMS of the left DLPFC has antidepressant effects in patients unresponsive to at least one pharmacological therapy. Additionally, these trials indicated an acute antidepressant effect of rTMS relative to placebo following attempts at pharmacological therapy (O’Reardon et al., 2007; George et al., 2010).

As stated in a recent study by Han and colleagues (2003), despite FDA approval of DLPFC-targeted rTMS for depression treatment 15 years ago, the precise mechanisms underlying its antidepressant effects remain elusive. To tackle this issue, the research team analyzed TMS-electroencephalogram (EEG) data from 64 healthy control (HC) subjects and 53 patients with major depressive disorder (MDD) pre- and post-rTMS treatment. The findings revealed that, prior to the treatment, patients with MDD exhibited lower activity in the DLPFC, hippocampus (HPC), and orbitofrontal cortex (OFC), as well as reduced DLPFC–OFC connectivity compared to HCs, as measured by the TMS-evoked potential (TEP) amplitude ratio of P60/N100, and local mean field amplitude area under the curve. Remarkably, post-active rTMS treatment, MDD patients showed significant increases in activity within the DLPFC, HPC, and OFC at the sensor level. Particularly, the enhancement in HPC activity through delta changes was closely associated with the alleviation of depressive outcome variables. The findings highlight the important role of the orbitofrontal–hippocampal pathway in the reduction of depressive symptoms following rTMS treatment, suggesting additional potential targets for brain stimulation in depression (Han et al., 2023).

Another large multicenter, placebo-controlled study also showed that deep TMS, a modified coil form with lower focality and thus higher penetration depth, as monotherapy was attempted in patients with 1–2 unsuccessful pharmacological treatments whose antidepressant effects were compared to placebo stimulation (Levkovitz et al., 2015). Interestingly, these effects were more pronounced in patients with lower (1–2 treatment attempts) and more serious (3–4 treatment attempts) treatment resistance and were stable over 12 weeks of maintenance therapy. The antidepressant efficacy of rTMS is also supported by several meta-analyses (Berlim et al., 2014) and evidence-based guidelines (Lefaucheur et al., 2014), in which 26 positive and 14 negative studies were identified, which show antidepressant efficacy at a high level of evidence.

Furthermore, the interplay between rTMS and antidepressant medication has been the subject of multiple studies, with a focus on understanding their combined efficacy in treating MDD. Rumi et al. (2005) and Fitzgerald et al. (2009) found that rTMS could function as a standalone treatment and enhance the effects of antidepressants. These findings were further substantiated in a meta-analysis by Gaynes et al. (2014), revealing increased remission rates when rTMS was combined with antidepressants.

Building on these studies, Wilke and colleagues (2022) conducted a retrospective analysis of patients with non-psychotic MDD undergoing rTMS treatment while on medication. The study focused on those using psychostimulants such as lisdexamfetamine/dextroamphetamine, methylphenidate/dexmethylphenidate, and modafinil/armodafinil. The findings indicated that patients taking psychostimulants experienced a significantly greater clinical improvement than those not on these medications during their rTMS treatment. The psychostimulant group showed significant enhancements in sleep and mood/cognition domains. Interestingly, smaller doses of lisdexamfetamine/dextroamphetamine correlated with better rTMS outcomes. Despite promising results, the authors cautioned that the study’s small sample size, limited data on psychostimulant use duration, and the lack of causal evidence necessitate further trials for solidifying the safety and efficacy of combining psychostimulants with rTMS (Wilke et al., 2022).

These studies underscore the promising potential of rTMS in both standalone and combined treatments for depression and the need for further research to fully understand the complexities and optimal applications of this therapy.

### rTMS in catatonia

In recent years, rTMS has received increasing attention as a therapeutic tool in treating psychiatric disorders and is even discussed as a treatment alternative to electroconvulsive therapy (Arns et al., 2012; Olbrich et al., 2015). Till date, the successful use of rTMS for catatonia was reported in nine cases (Chung et al., 2018). Most reports showed an impressive effect. In this context, the practical evidence for the use of rTMS in the treatment of catatonia is particularly noteworthy, especially in consideration of the assumed mechanism in catatonia. Recent reviews highlight the hyperactivity of premotor areas as an important pathophysiological feature of catatonia (Leuchter et al., 2013), essential in addressing motor system pathology in schizophrenia.

Although disturbances in neural maturation during the onset of schizophrenia suggest the presence of hypokinetic motor irregularities and potential malfunctions within the cerebral motor network associated with catatonia, a more comprehensive understanding is needed. Additional discussions have posited a decreased activity in the DLPFC and other areas of the brain. Nevertheless, contemporary consensus seems to gravitate toward hyperactivity within the pre-supplementary motor area (pre-SMA) and the supplementary motor area (SMA) as the principal causative factors underlying catatonia (Ding et al., 2014; Leuchter et al., 2015; Sadaghiani and Kleinschmidt, 2016). Moreover, interventions that inhibit the SMA have demonstrated efficacy in ameliorating psychomotor retardation in patients afflicted with severe mental illness (Sadaghiani and Kleinschmidt, 2016).

Presently, the empirical support for utilizing rTMS as a therapeutic intervention for catatonia remains somewhat restricted to a series of case studies. Even though the pre-ponderance of these studies consists of relatively few cases, there is an emerging body of evidence suggesting that rTMS could be particularly potent in cases of refractory schizophrenic catatonia.

To further consolidate these preliminary findings, there is an imperative need to conduct more extensive research. Future inquiries should focus on specific combinations of rTMS with pharmacotherapy or psychotherapy. Furthermore, understanding the neurobiological predictors of response, differential indications, and maintenance treatment could be invaluable in advancing this treatment modality, ultimately enhancing patient care and clinical outcomes.

### rTMS and sleep modulation

Although rTMS has been initially developed as a therapeutic tool for neurological and psychiatric disorders, it is now gaining progressive momentum in the area of sleep research (Hallett, 2007; Saebipour et al., 2015). Sleep, as vital physiological process, is intricately connected to cognitive function and overall health (Walker, 2009).

Gorgoni et al. (2016) investigated the effects of rTMS on sleep in healthy subjects. They found that a single session of slow (1 Hz) rTMS over the DLPFC before sleep increased the amount of slow-wave sleep (SWS) and the slow-wave activity (SWA) during subsequent sleep, especially in the first sleep cycle. They also observed that rTMS caused changes in the functional connectivity between several brain regions during sleep (Gorgoni et al., 2016).

Saebipour et al. (2015) demonstrated that rTMS could be used to improve sleep quality. They used 20 Hz rTMS over the DLPFC in healthy subjects and observed improved sleep quality and increased total sleep time (Saebipour et al., 2015).

Centorino et al. (2020) examined the interplay between rTMS treatment, sleep, and neural plasticity. The results suggest that high sleep quality facilitates plasticity and learning, subsequently improving the effectiveness of rTMS. However, the influence of high-frequency and low-frequency rTMS on sleep remains inconclusive. They further highlighted that certain sleep characteristics, such as total sleep time and sleep continuity, are potentially important, yet no sufficient determinants for the homeostatic plasticity were induced by SWS (Centorino et al., 2020).

Ultimately, research on the impact of rTMS on sleep, particularly in healthy individuals, is still in its early stages. Determining the optimal rTMS parameters, understanding individual differences in response, and elucidating the underlying neural mechanisms remain the ongoing areas of investigation with potential implications for populations with disordered sleep.

### rTMS in insomnia and MCI

The physiology of the sleep–wake cycle is characterized by cyclic oscillations of EEG activity, which are divided into non-rapid eye movement (NREM) and rapid eye movement (REM) sleep stages. Starting in the 1970s, numerous research studies began investigating the relationship between the K-complex and the delta waves of NREM sleep and the relationship between the K-complex and sleep cyclicity as a complex multifunctional phenomenon of the sleeping brain involved in information processing (Halász, 2005).

Building on this understanding of NREM sleep and the intricate dynamics of brain activity it involves, more recent research has focused on the role of slow cortical oscillations (SO; 0.5–1 Hz) and thalamocortical spindle activity (12–15 Hz) during NREM sleep. This coupling is considered crucial for memory formation. Indeed, various neuromodulation techniques using slow oscillatory transcranial stimulation have been demonstrated to enhance functional cross-frequency coupling between memory-relevant brain oscillations. Notably, they have been shown to improve visual memory consolidation in patients with mild cognitive impairment (MCI), a precursor to Alzheimer’s disease (Ladenbauer et al., 2017). This beneficial impact on the cognitive function suggests the far-reaching potential of these techniques in neurophysiological modulation.

In a separate but related development, techniques such as transcranial magnetic, electrical, and auditory stimulation are being applied in the treatment of sleep disorders. These methods have demonstrated utility in modulating arousal and sleep patterns, particularly in patients suffering from insomnia and hypersomnia, with implications for the broader improvement of mental health.

Individuals suffering from insomnia typically exhibit persistent hyperarousal over a 24-h cycle, as well as a decrease in SWS and an increase in power of fast frequencies (within the EEG β-range) during NREM sleep (Spiegelhalder et al., 2012). Remarkably, even during the deepest sleep stages, sensory and sensorimotor regions appear to maintain a relative level of activity in insomnia patients compared to controls and the remainder of the sleeping brain (Brody et al., 1999). It has been reported that the utilization of non-invasive brain stimulation (NIBS) techniques for neuromodulation in insomnia patients can enhance slow oscillations (Geiser et al., 2020). Future investigations are warranted to evaluate the clinical implications of these slow oscillations, which may hold promise for ameliorating complaints of non-restorative sleep. The hyperarousal model of primary insomnia posits that impaired attenuation of arousal during sleep may be the root of experiencing non-restful sleep. Examination of EEG spectral power values for standard frequency bands during sleep in 25 patients with primary insomnia and 29 controls with reported good sleep showed that patients with primary insomnia had significantly increased spectral power values in the EEG beta and sigma frequency bands during phase 2 of NREM sleep. This result suggests that EEG beta activity is a marker of cortical arousal, and EEG sleep spindle activity (sigma) is an index of sleep-related protective mechanisms (Spiegelhalder et al., 2012). These findings underscore the role of arousal dysregulation in sleep disturbances.

A recent pilot study by Holbert et al. (2023) on 20 primary insomnia patients utilizing bifrontal low-frequency (LF) TMS demonstrated significant improvements in subjective sleep outcome scores and symptom severity. However, no sham control data were recorded (Holbert et al., 2023). These findings are in line with a recent review in the context of the potential of rTMS in the treatment of sleep disorders provided by Oroz and colleagues (2021). While subjective sleep improvement is commonly reported across various studies post-rTMS, objective improvements remain inconsistent. Notably, they reported that only few studies were sham-controlled, a factor to be considered for potential placebo effects. However, among these, the placebo effect of rTMS varied significantly (Oroz et al., 2021).

Extrapolating from these findings, it becomes evident that the capacity to modulate sleep dynamics could have profound implications in psychiatric research and clinical practice. As our understanding of the complexities of sleep and its impact on cognitive and behavioral processes deepens, the prospective therapeutic benefits of sleep modulation across various mental disorders increasingly stand out as a pivotal area of research exploration.

## Discussion

In the treatment of depression, partial and non-responses to rTMS are common, creating an ongoing quest to understand why responses to this neuromodulation treatment differ among patients (Li et al., 2022). This challenge has inspired an extensive exploration of brain imaging data, with neuroimaging and neurophysiological measures suggested as promising biomarker candidates (Brakemeier et al., 2007; Arns et al., 2010).

In this regard, various studies have identified potential predictors of antidepressant response in structural alterations of both gray and white matter, particularly the reduced gray matter volumes in the insula, anterior cingulate, and the HPC (Li et al., 2022). Interestingly, the anterior cingulate cortex has been implicated in the treatment response of diverse conditions including major depression, obsessive-compulsive disorder, generalized anxiety, and post-traumatic stress disorders (Lisanby et al., 2008; Bares et al., 2009; Garnaat et al., 2019). This suggests a generalized role for this area in predicting treatment response across conditions.

In addition, a recent cross-diagnostic meta-analysis conducted by Kan et al. (2023) demonstrated the efficacy of rTMS targeted at the left DLPFC in treating a wide range of neuropsychiatric symptoms. However, beyond the anterior cingulate cortex, less is known about the role of other brain regions in treatment response. For instance, studies have hinted that pretreatment hypometabolism and altered functional connectivity within certain regions, such as the OFC, may correlate with the antidepressant response of rTMS (Herwig et al., 2007; Sienaert et al., 2014).

In addition to the growing body of research that highlights the role of specific brain regions and their structural alterations in predicting the treatment response, there is also emerging evidence on the potential of neurophysiological measures serving as biomarkers. For instance, Canali et al. (2017) have illuminated this possibility in the context of bipolar disorder (BD). Through a combination of TMS and EEG, they discovered a consistent reduction in natural frequencies in BD patients. Importantly, this reduction was present regardless of treatment response or the clinical status of patients, suggesting that these neurophysiological measures could offer a stable, disease-specific biomarker.

As we pivot toward a more personalized approach in psychiatric treatment, these findings gain significance. The burgeoning field of personalized medicine in psychiatry necessitates the identification of robust biomarkers that can reflect the function of the central nervous system at the neuronal activity level (Olbrich et al., 2015). These biomarkers would be instrumental in predicting the response to specific treatments and monitoring changes post-treatment.

In this context, rTMS stands out due to its proven efficacy and its potential for individualized treatment approaches. Research has demonstrated the predictability of treatment response to rTMS based on brain activity patterns and the association of post-treatment changes in these patterns with the overall treatment response, particularly in depression (Hansbauer et al., 2020). This suggests that rTMS could be tailored to individual patient needs, thereby catering to various psychiatric conditions including MDD, catatonia, and insomnia (Garnaat et al., 2019). Therefore, the convergence of neurophysiological biomarkers and personalized treatment approaches such as rTMS could pave the way for a new era in psychiatric care. Future research aims to identify patient-specific factors to guide rTMS treatment decisions. Potential biomarkers to be evaluated range from morphological and neurophysiological brain abnormalities to genetic factors and biomarkers derived from neuroimaging and EEG. The validation of imaging and electrophysiological biomarkers associated with rTMS is crucial, as is the prospective evaluation of clinical predictors and personalized patient response to rTMS.

The use of rTMS to trigger slow waves in humans holds promise for personalized treatment approaches. The elicitation of slow waves via TMS, dependent on the dosage and administration site, can significantly increase SWA across the scalp, possibly inducing a shift to a deep sleep stage (Massimini et al., 2007). This finding paves the way for larger studies investigating the effect of rTMS on deep sleep induction, a state critical to mental and physical health (Richter et al., 2017).

In summary, the application of rTMS to mental health disorders provides exciting opportunities for personalized treatment approaches, particularly through the identification and use of neurophysiological and neuroimaging biomarkers. As we continue to understand the role of brain activity in neuropsychiatric disorders, rTMS stands as a potential game-changer, enabling clinicians to tailor treatment strategies for individual patients and ultimately enhance treatment outcomes.

## References

[B1] Arns M. Drinkenburg W. H. Fitzgerald P. B. Kenemans J. L. (2012). Neurophysiological predictors of non-response to rTMS in depression. Brain Stimul. 5 (4), 569–576. 10.1016/j.brs.2011.12.003 22410477

[B2] Arns M. Spronk D. Fitzgerald P. B. (2010). Potential differential effects of 9 Hz rTMS and 10 Hz rTMS in the treatment of depression. Brain Stimul. 3 (2), 124–126. 10.1016/j.brs.2009.07.005 20633440

[B3] Bares M. Kopecek M. Novak T. Stopkova P. Sos P. Kozeny J. (2009). Low frequency (1-hz), right prefrontal repetitive transcranial magnetic stimulation (rTMS) compared with venlafaxine er in the treatment of resistant depression: A double-blind, single-centre, randomized study. J. Affect. Disord. 118 (1–3), 94–100. 10.1016/j.jad.2009.01.032 19249105

[B4] Berlim M. T. van den Eynde F. Tovar-Perdomo S. Daskalakis Z. J. (2014). Response, remission and drop-out rates following high-frequency repetitive transcranial magnetic stimulation (rTMS) for treating major depression: A systematic review and meta-analysis of randomized, double-blind and sham-controlled trials. Psychol. Med. 44 (2), 225–239. 10.1017/s0033291713000512 23507264

[B5] Brakemeier E.-L. Luborzewski A. Danker-Hopfe H. Kathmann N. Bajbouj M. (2007). Positive predictors for antidepressive response to prefrontal repetitive transcranial magnetic stimulation (rTMS). J. Psychiatric Res. 41 (5), 395–403. 10.1016/j.jpsychires.2006.01.013 16554071

[B6] Brody A. L. Saxena S. Silverman D. H. Alborzian S. Fairbanks L. A. Phelps M. E. (1999). Brain metabolic changes in major depressive disorder from pre-to post-treatment with paroxetine. Psychiatry Res. Neuroimaging 91 (3), 127–139. 10.1016/s0925-4927(99)00034-7 10641577

[B8] Canali P. Casarotto S. Rosanova M. Sferrazza-Papa G. Casali A. G. Gosseries O. (2017). Abnormal brain oscillations persist after recovery from bipolar depression. Eur. Psychiatry J. Assoc. Eur. Psychiatrists 41, 10–15. 10.1016/j.eurpsy.2016.10.005 28049075

[B62] Centorino M. B. Bajor L. A. Gootam P. K. Nakase-Richardson R. Kozel F. A. (2020). The relationship of transcranial magnetic stimulation with sleep and plasticity. J. psychiatric practice 26 (6), 434–443. 10.1097/PRA.0000000000000506 33275381

[B9] Chung S. W. Sullivan C. M. Rogasch N. C. Hoy K. E. Bailey N. W. Cash R. F. H. (2018). The effects of individualised intermittent theta burst stimulation in the prefrontal cortex: A TMS-EEG study. Hum. Brain Mapp. 40 (2), 608–627. 10.1002/hbm.24398 30251765 PMC6865598

[B10] Ding L. Shou G. Yuan H. Urbano D. Cha Y. H. (2014). Lasting modulation effects of rTMS on neural activity and connectivity as revealed by resting-state EEG. IEEE Trans. Biomed. Eng. 61 (7), 2070–2080. 10.1109/tbme.2014.2313575 24686227 PMC5638649

[B61] Fitzgerald P. B. Hoy K. McQueen S. Maller J. J. Herring S. Segrave R. (2009). A randomized trial of rTMS targeted with MRI based neuro-navigation in treatment-resistant depression. Neuropsychopharmacology: Official publication of the American College of Neuropsychopharmacology 34 (5), 1255–1262. 10.1038/npp.2008.233 19145228

[B12] Garnaat S. L. Fukuda A. M. Yuan S. Carpenter L. L. (2019). Identification of clinical features and biomarkers that may inform a personalized approach to rTMS for depression. Personalized Med. Psychiatry 17–18, 4–16. 10.1016/j.pmip.2019.09.001 PMC8094130 33954269

[B13] Geiser T. Hertenstein E. Fehér K. Maier J. G. Schneider C. L. Züst M. A. (2020). Targeting arousal and sleep through noninvasive brain stimulation to improve mental health. Neuropsychobiology 79 (4–5), 284–292. 10.1159/000507372 32408296

[B14] George M. S. Lisanby S. H. Avery D. McDonald W. M. Durkalski V. Pavlicova M. (2010). Daily left prefrontal transcranial magnetic stimulation therapy for major depressive disorder: A sham-controlled randomized trial. Archives General Psychiatry 67 (5), 507–516. 10.1001/archgenpsychiatry.2010.46 20439832

[B15] George M. S. Lisanby S. H. Sackeim H. A. (1999). Transcranial magnetic stimulation: Applications in neuropsychiatry. Archives General Psychiatry 56 (4), 300–311. 10.1001/archpsyc.56.4.300 10197824

[B16] Goldsworthy M. R. Hordacre B. Rothwell J. C. Ridding M. C. (2021). Effects of rTMS on the brain: Is there value in variability? Cortex 139, 43–59. 10.1016/j.cortex.2021.02.024 33827037

[B17] Gorgoni M. Lauri G. Truglia I. Cordone S. Sarasso S. Scarpelli S. (2016). Comparisons among different cortical targets and stimulation frequencies in sleep EEG modulation induced by rTMS. J. Psychophysiol. 30 (3), 101–113. 10.1027/0269-8803/a000161

[B18] Halász P. (2005). K-Complex, a reactive EEG graphoelement of NREM sleep: An old chap in a new garment. Sleep. Med. Rev. 9 (5), 391–412. 10.1016/j.smrv.2005.04.003 16122950

[B19] Hallett M. (2007). Transcranial magnetic stimulation: A primer. Neuron 55 (2), 187–199. 10.1016/j.neuron.2007.06.026 17640522

[B20] Han S. Li X. X. Wei S. Zhao D. Ding J. Xu Y. (2023). Orbitofrontal cortex-hippocampus potentiation mediates relief for depression: A randomized double-blind trial and TMS-EEG study. Medicine 4 (6), 101060. 10.1016/j.xcrm.2023.101060 37263267 PMC10313932

[B21] Hansbauer M. Wagner E. Strube W. Röh A. Padberg F. Keeser D. (2020). rTMS and tDCS for the treatment of catatonia: A systematic review. Schizophrenia Res. 222, 73–78. 10.1016/j.schres.2020.05.028 32600779

[B23] Herwig U. Fallgatter A. J. Höppner J. Eschweiler G. W. Kron M. Hajak G. (2007). Antidepressant effects of augmentative transcranial magnetic stimulation: Randomised multicentre trial. Br. J. Psychiatry 191 (5), 441–448. 10.1192/bjp.bp.106.034371 17978325

[B24] Holbert R. C. Carr B. R. Bussing R. (2023). An open label pilot trial of sequential bifrontal low frequency r-TMS in the treatment of primary insomnia. Psychiatry Res. 324, 115194. 10.1016/j.psychres.2023.115194 37054553 PMC11287436

[B25] Kan R. L. D. Padberg F. Giron C. G. Lin T. T. Z. Zhang B. B. B. Brunoni A. R. (2023). Effects of repetitive transcranial magnetic stimulation of the left dorsolateral prefrontal cortex on symptom domains in neuropsychiatric disorders: A systematic review and cross-diagnostic meta-analysis. Lancet Psychiatry 10 (4), 252–259. 10.1016/S2215-0366(23)00026-3 36898403

[B26] Ladenbauer J. Ladenbauer J. Külzow N. de Boor R. Avramova E. Grittner U. (2017). Promoting sleep oscillations and their functional coupling by transcranial stimulation enhances memory consolidation in mild cognitive impairment. J. Neurosci.: the official journal of the Society for Neuroscience 37 (30), 7111–7124. 10.1523/JNEUROSCI.0260-17.2017 PMC6705731 28637840

[B27] Lefaucheur J.-P. Aleman A. Baeken C. Benninger D. H. Brunelin J. Di Lazzaro V. (2020). Evidence-based guidelines on the therapeutic use of repetitive transcranial magnetic stimulation (rTMS): An update (2014–2018). Clin. Neurophysiol. 131 (2), 474–528. 10.1016/j.clinph.2019.11.002 31901449

[B28] Lefaucheur J.-P. André-Obadia N. Antal A. Ayache S. S. Baeken C. Benninger D. H. (2014). Evidence-based guidelines on the therapeutic use of repetitive transcranial magnetic stimulation (rTMS). Clin. Neurophysiol. 125 (11), 2150–2206. 10.1016/j.clinph.2014.05.021 25034472

[B29] Leuchter A. F. Cook I. A. Feifel D. Goethe J. W. Husain M. Carpenter L. L. (2015). Efficacy and safety of low-field synchronized transcranial magnetic stimulation (sTMS) for treatment of major depression. Brain Stimul. 8 (4), 787–794. 10.1016/j.brs.2015.05.005 26143022

[B30] Leuchter A. F. Cook I. A. Jin Y. Phillips B. (2013). The relationship between brain oscillatory activity and therapeutic effectiveness of transcranial magnetic stimulation in the treatment of major depressive disorder. Front. Hum. Neurosci. 7, 37. 10.3389/fnhum.2013.00037 23550274 PMC3581824

[B31] Levkovitz Y. Isserles M. Padberg F. Lisanby S. H. Bystritsky A. Xia G. (2015). Efficacy and safety of deep transcranial magnetic stimulation for major depression: a prospective multicenter randomized controlled trial. World Psychiatry: official journal of the World Psychiatric Association (WPA) 14 (1), 64–73. 10.1002/wps.20199 25655160 PMC4329899

[B32] Li M. Zhu Y. Zhang X. Yang H. Zhang S. Liu J. (2022). 1Hz rTMS over left DLPFC rewired the coordination with hippocampus in insomnia patients: A pilot study. Brain Stimul. 15 (2), 437–440. 10.1016/j.brs.2022.02.011 35219925

[B33] Licht C. Alt E. Fuchs S. Ruttmann A. Richter K. Hillemacher T. (2021). Repetitive transcranial magnetic stimulation (rTMS) for catatonia– a case report. Brain Stimul. 14 (6), 1636. 10.1016/j.brs.2021.10.154

[B34] Lisanby S. H. Husain M. M. Rosenquist P. B. Maixner D. Gutierrez R. Krystal A. (2008). Daily left prefrontal repetitive transcranial magnetic stimulation in the acute treatment of major depression: Clinical predictors of outcome in a multisite, randomized controlled clinical trial. Neuropsychopharmacology 34 (2), 522–534. 10.1038/npp.2008.118 18704101

[B36] Massimini M. Ferrarelli F. Esser S. K. Riedner B. A. Huber R. Murphy M. (2007). Triggering sleep slow waves by transcranial magnetic stimulation. Proc. Natl. Acad. Sci. 104 (20), 8496–8501. 10.1073/pnas.0702495104 17483481 PMC1895978

[B38] Olbrich S. van Dinteren R. Arns M. (2015). Personalized medicine: Review and perspectives of promising baseline EEG biomarkers in major depressive disorder and attention deficit hyperactivity disorder. Neuropsychobiology 72 (3–4), 229–240. 10.1159/000437435 26901357

[B39] O’Reardon J. P. Solvason H. B. Janicak P. G. Sampson S. Isenberg K. E. Nahas Z. (2007). Efficacy and safety of transcranial magnetic stimulation in the acute treatment of major depression: A multisite randomized controlled trial. Biol. Psychiatry 62 (11), 1208–1216. 10.1016/j.biopsych.2007.01.018 17573044

[B40] Oroz R. Kung S. Croarkin P. E. Cheung J. (2021). Transcranial magnetic stimulation therapeutic applications on sleep and insomnia: A review. Sleep Sci. Pract. 5 (3), 3. 10.1186/s41606-020-00057-9

[B41] Pashut T. Wolfus S. Friedman A. Lavidor M. Bar-Gad I. Yeshurun Y. (2011). Mechanisms of magnetic stimulation of central nervous system neurons. PLoS Comput. Biol. 7 (3), e1002022. 10.1371/journal.pcbi.1002022 21455288 PMC3063755

[B42] Peng Z. Zhou C. Xue S. Bai J. Yu S. Li X. (2018). Mechanism of repetitive transcranial magnetic stimulation for depression. Shanghai Archives Psychiatry 30 (2), 84–92. 10.11919/j.issn.1002-0829.217047 PMC5936045 29736128

[B45] Reithler J. Peters J. C. Sack A. T. (2011). Multimodal transcranial magnetic stimulation: Using concurrent neuroimaging to reveal the neural network dynamics of noninvasive brain stimulation. Prog. Neurobiol. 94 (2), 149–165. 10.1016/j.pneurobio.2011.04.004 21527312

[B46] Richter K. Acker J. Miloseva L. Peter L. Niklewski G. (2017). Management of chronic tinnitus and insomnia with repetitive transcranial magnetic stimulation and cognitive behavioral therapy – A combined approach. Front. Psychol. 8, 575. 10.3389/fpsyg.2017.00575 28484405 PMC5399016

[B48] Rumi D. O. Gattaz W. F. Rigonatti S. P. Rosa M. A. Fregni F. Rosa M. O. (2005). Transcranial magnetic stimulation accelerates the antidepressant effect of amitriptyline in severe depression: A double-blind placebo-controlled study. Biol. Psychiatry 57 (2), 162–166. 10.1016/j.biopsych.2004.10.029 15652875

[B49] Sadaghiani S. Kleinschmidt A. (2016). Brain networks and α-oscillations: Structural and functional foundations of cognitive control. Trends Cognitive Sci. 20 (11), 805–817. 10.1016/j.tics.2016.09.004 27707588

[B50] Saebipour M. R. Joghataei M. T. Yoonessi A. Sadeghniiat-Haghighi K. Khalighinejad N. Khademi S. (2015). Slow oscillating transcranial direct current stimulation during sleep has a sleep-stabilizing effect in chronic insomnia: a pilot study. J. sleep research 24 (5), 518–525. 10.1111/jsr.12301 26014344

[B51] Sienaert P. Dhossche D. M. Vancampfort D. De Hert M. Gazdag G. (2014). A clinical review of the treatment of catatonia. Front. Psychiatry 5, 181. 10.3389/fpsyt.2014.00181 25538636 PMC4260674

[B52] Spiegelhalder K. Regen W. Feige B. Holz J. Piosczyk H. Baglioni C. (2012). Increased EEG sigma and beta power during NREM sleep in primary insomnia. Biol. Psychol. 91 (3), 329–333. 10.1016/j.biopsycho.2012.08.009 22960269

[B54] Walker M. P. (2009). The role of sleep in cognition and emotion. Ann. N. Y. Acad. Sci. 1156 (1), 168–197. 10.1111/j.1749-6632.2009.04416.x 19338508

[B58] Wassermann E. M. (1998). Risk and safety of repetitive transcranial magnetic stimulation: Report and suggested guidelines from the international workshop on the safety of repetitive transcranial magnetic stimulation, june 5–7, 1996. Electroencephalogr. Clin. Neurophysiology/Evoked Potentials Sect. 108 (1), 1–16. 10.1016/s0168-5597(97)00096-8 9474057

[B59] Wilke S. A. Johnson C. L. Corlier J. Marder K. G. Wilson A. C. Pleman C. M. (2022). Psychostimulant use and clinical outcome of repetitive transcranial magnetic stimulation treatment of major depressive disorder. Depress. Anxiety 39 (5), 397–406. 10.1002/da.23255 35389536

[B60] Yip A. G. George M. S. Tendler A. Roth Y. Zangen A. Carpenter L. L. (2017). 61% of unmedicated treatment resistant depression patients who did not respond to acute TMS treatment responded after four weeks of twice weekly deep TMS in the Brainsway pivotal trial. Brain Stimul. 10 (4), 847–849. 10.1016/j.brs.2017.02.013 28330592

